# A Biographical Sketch of the Professional Life and Character of the Late Henry Huntt, M. D., of Washington City, D. C.

**Published:** 1838-11-07

**Authors:** T. Miller

**Affiliations:** Chairman of a Committee appointed by the Medical Association of Washington, &c. &c.


### A BIOGRAPHICAL SKETCH *of the Professional Life and Character of the late* Henry Huntt, M. D., *of Washington City, D. C.* By T. Miller, M. D., Chairman of a Committee appointed by the Medical Association of Washington, &c. &c.

[Published by the direction of the Association.]

It is due to eminent worth in every class of life, that it should not be suffered to sink into the tomb without some more permanent tribute of respect, than a passing obituary notice; but the lives of those who belong to what are termed the learned professions, generally afford but scanty materials for a biography; and when, as in the case of the lamented subject of this brief notice, that aversion to egotism which usually accompanies merit, does not permit the individual to make his own life and actions a frequent topic of discourse, even among his most intimate friends, the record must be still more meagre and unsatisfactory.

Henry Huntt was a native of Calvert county, Maryland, where he received his early education. When about eighteen years of age, he left his parental roof to reside with his uncle, Doctor Clement Smith, a most respectable and talented practitioner of medicine in Prince George's county, Maryland. Shortly afterwards he entered his uncle's office as a student of medicine, and soon, by his urbanity of manners and assiduous attention to his studies, he gained the esteem and friendship of all who knew him. Early in life he exhibited dawnings of future professional eminence, while yet a student; and before he had read medicine eighteen months, he was intrusted by his uncle with the entire treatment of patients, whose confidence he soon possessed.

In 1805 and '6, he attended a course of medical lectures in the University of Pennsylvania; and by his studious habits and close attention to the lectures, gained the regard and approbation of nearly all the professors of that day, particularly that of the venerated father of American surgery, which he enjoyed to the death of that gifted and honoured man. On his return to Maryland in the spring of 1806, he entered into partnership in practice with his uncle, and soon became distinguished by his attention and kindness to his patients. He was particularly so, for his success in the treatment of the diseases of children, and many parents of the present day speak with gratitude and praise of his skilful efforts in preserving their innocent offspring from an untimely grave. In 1808 and '9, he made experimental observations on the character of the discharges in dysentery and diarrhœa—the chronic forms of those diseases more particularly—a subject which has lately attracted the attention of the medical experimenters and speculators, which led him to the practice of substituting an acid for a mercurial and alkaline treatment of those diseases.

A country practice affording too limited a field of professional preferment and emolument for one of Huntt's ambitious views, he, amid the regrets of a large circle of friends, abandoned his practice in Prince George's, and came to the city of Washington in the fall of 1810, with the intention of applying for the situation of surgeon's mate in the U. S. navy. This he desired, that he might have an opportunity of extending his medical views and experience. He succeeded in being appointed on the 2d June, 1811, and performed the duties incident to his station for more than two years, in a manner creditable to himself and satisfactory to his superiors. Considerations of a private nature induced him to resign this appointment on the 31st August, 1813; and he had determined to enter into private practice in Washington, when, in 1814, a more than usual demand for hospital surgeons in our army existing, he applied, and was appointed by Mr. Armstrong on the 17th March, 1814, to take charge of the Burlington Hospital, Vermont, to which he immediately repaired. In a short time, by his indefatigable exertions, untiring zeal and promptness, he attained rank in the "highest grade of surgeons," performing many important surgical operations, among which we may mention the operation on the shoulder joint of Lieut. Duncan, of the U. S. navy, an account of which is given in the Medical Recorder for 1828.

At the same time that he gained the esteem and friendship of his brother officers generally, his devotion to his official duties is best testified to by Dr. Mann, his able predecessor at Burlington, who thus speaks of him:—"To Dr. Huntt, who succeeded me at this post, the most liberal encomiums are due; my acquaintance with him gave me an opportunity more fully to appreciate his merits as a director of that establishment, which, from its infancy to the close of the campaign, had a claim to pre-eminence." While at Burlington, his successful treatment of the pneumonia then raging there, together with his reports of cases, those of the effects of the acetate of lead in various diseases particularly, deserve to be most honourably mentioned. At the close of the war in 1815, Dr. Huntt returned to the District of Columbia, and with a number of the officers of the army was disbanded on the 1st June, 1815. In leaving the army, he felt conscious of having acquitted himself creditably to his well-earned reputation, and of having performed useful services to his country, a conviction sustained by more than one of those who had braved with him the brunt of that eventful and arduous campaign. He now permanently established himself in Washington city, and once more entered the arena of private practice. Soon he became distinguished, taking a stand high among the physicians of that day; with the view of attaining greater proficiency in the practice of medicine, he abandoned that of surgery and obstetrics, and devoted himself exclusively to therapeutics and pathology, and few can boast of having been more successful than he was. Soon after he settled in Washington, in consequence of his success in the treatment of the epidemic pneumonia, which at that time pervaded nearly the whole of this section of country, and proved almost universally fatal, he, at the request of many friends, published a brief view of the pathology and treatment of that disease, the general adoption of which led to a much less fatal result in the treatment, for which he received many flattering public acknowledgments.

But a few years elapsed after he settled in Washington, before he enjoyed a most respectable and lucrative practice. By his deportment and conduct, both private and professional, he soon gained the esteem of his seniors in the profession, and the confidence of his patients. About the year 1820, he originated, on his individual responsibility, a health office in Washington, and was for several years, under the sanction of the corporation, the only acting officer. After a few years, he succeeded in having organized a regular Board of Health, of which, in 1824, he was elected President. This situation he would have retained as long as he lived, had not the great increase of his private and professional engagements, together with the delicate state of his health, rendered it advisable for him to retire from it, which he did in 1833. During the whole period that he held this situation, he always kept in view the comfort and welfare of his fellow-citizens, deporting himself with dignity and firmness. In 1832, when the district was visited by the epidemic cholera, he displayed most particularly his zeal for the public good;—though engaged in a most arduous practice, with many unforeseen difficulties to encounter, he acted as President of the Board of Health with promptness and firmness, at the same time that he consented to perform the duties of one of the consulting physicians to the several hospitals in the city. He never ceased his efforts day or night to render aid and consolation to his fellow-citizens; and to the great fatigue and exposure he necessarily encountered during that season of distress and desolation, we may date the commencement of the disease which ultimately terminated his existence.

In the year 1832, he wrote, from data furnished by a distinguished medical friend from Virginia, a short treatise on the virtues of the waters of the Red Sulphur Springs, in Virginia, in consumption, &c., &c. Little did he suppose at that time, it would have been his lot, so soon to test their virtues on himself. In the fall of 1836, though his health had been previously much impaired, he first discovered symptoms of pulmonary disease; he was attacked with slight cough, soon attended with bloody expectoration, not sufficiently severe, however, to prevent him from pursuing his professional avocation. In the spring of 1837, his mind being greatly distressed by the loss of an only brother, to whom he was much attached, he was seized with slight hæmorrhage, succeeded by hectic chills and other symptoms indicating pulmonary and mesenteric disease. As time diminished the acute pangs of grief occasioned by his late family bereavement, and as the general warmth of the summer approached, his health and spirits became better, and he was induced to accept the appointment of visiter and medical inspector of the Military Academy at West Point. On his return home in July, his health much injured by the northern climate, he determined by the advice of Dr. Physick to take some relaxation from his labours, and endeavour to repair his lost energies, both of body and mind. With this object he visited the Red Sulphur Springs, so long his favorite theme, resolved to give them a fair trial in his own case, at the same time that he made observations of the effects of the waters on others. He remained at these springs, collecting notes and certificates corroborative of his views, till the last of August, when he returned apparently very much benefited in his health, which continued sufficiently good to enable him to resume his professional pursuits, till the commencement of November, when, from imprudent exposure at night, his lungs became re-excited, which caused a return of all his former unpleasant symptoms, and confined him to the house nearly the whole of the winter of 1837 and '38: during this time he was occupied with private consultations, and arranging his notes on the Red Sulphur Springs for publication. In the spring of 1838, he published the result of his observations in a pamphlet, entitled "A Visit to the Red Sulphur Springs of Virginia, &c, &c.;" convinced that he was conferring a benefit on mankind by calling attention to the happy effect of the water of these springs in several hopeless diseases. Whether his impressions will be confirmed by the test of experience, must be determined by the results of future observations. It is to be regretted that Dr. Huntt did not rely on the stethescope in forming his opinions of the cases of *tubercular* phthisis said to be cured by those waters; he certainly evinced his own faith in their curative powers, for early in June, greatly reduced by disease, he set out with his family to visit once more the Red Sulphur Springs, where he remained until the 1st of Sempteber, when he was brought home by the judicious efforts only of a faithful and untiring friend, Gen. George Gibson, greatly emaciated, and evidently just approaching his end, but still hoping that his case was curable; that he now laboured only under the effects of debility, which would soon be removed by tonics; a hope which had been excited by the opinion of an eminent medical friend, whom he had met and consulted at the springs. This delusion lasted but a few days; soon he discovered that his powers were sinking; his cough, expectoration, together with prolonged and agonizing attacks of *singultus* were symptoms of too marked a character to escape the observation of a man so skilled in disease and death-bed scenes. He now saw his life was about to terminate, and gave directions relative to his family and the disposition of his remains. He always entertained a horror of his body undergoing the usual dressings, and of being buried alive; he therefore directed that as soon as the breath left the body, it should be covered over and not disturbed until it was to be placed in the coffin; that it should be kept until symptoms of decay were apparent; together with other directions similar to those given by Dr. Physick. He died on the 21st September, at 3 o'clock P. M., in the 56th year of his age.

As a physician Dr. Huntt was justly celebrated; his success in the treatment of many diseases, as fevers, infantile diseases, scarlatina, and epilepsy, entitled him to merited distinction. In the two latter diseases, he was very frequently consulted from a distance. Though the advantages of his early education were limited, he had by close attention and laborious study stored a mind, naturally good, with much valuable information. His judgment and discernment were of a high order; few men were ever more gifted in those respects. His mind was a storehouse of facts. He never lost a useful hint in his profession for want of observation. In the sick-room nothing ever escaped his keen and watchful eye; he seldom forgot what he thus obtained. In his directions he was celebrated for his minuteness and distinctness; he left nothing of importance to the discretion of nurses and attendants. To his patients his visits were always cheering, and he possessed the happy faculty of conducting himself, in every case, in such a manner as to inspire confidence at once; his knowledge of human nature taught him when to be abrupt and when to be mild in his intercourse with his patients. Though he had the reputation of being rough, even rude, he possessed the kindest and most sympathizing feelings for the sick, and by this adaptation of manner, he often dispelled the gloom so frequently a barrier to speedy convalescence. He always paid short visits to his patients, only long enough to understand their cases fully and prescribe for them, and never visited them unless absolutely necessary in his opinion. To this deportment, he has often said, he was indebted for much of his success in his profession.

From circumstances, not under his control, he never obtained regularly the degree of M. D., though in consideration of his eminence it was conferred on him by some of our most respectable universities, besides being constituted honorary member of several medical institutions of Europe. He was always a friend to science, and contributed much to the originating and sustaining many literary and medical societies. He was a member of the celebrated Φ. B. K. Society, the views and objects of which he sustained to the last. He was among the founders of the Columbian Institute, Medical Society of the District of Columbia, and the Medical Association of Washington. To each of these he was a most zealous supporter. He was a most uncompromising enemy to professional strife, which he considered as degrading to professional character, which he was ever ambitious to see attain the highest elevation. He had no professional jealousies to gratify, he was therefore liberal, candid and considerate to his brethren, all of whom viewed him as a friend; the seniors as an able coadjutor, the juniors as a counsellor. To the latter he was particularly considerate; he was never known to present an obstacle to the rise of youthful talent, or to repress laudable youthful ambition; to adopt the language applied to a most distinguished medical man of another country, "Few men ever maintained in the circle in which they practised, more respect and confidence from their professional brethren, or a higher character with the public as skilful physicians."

In consultation, Dr. Huntt displayed a penetration in the diagnosis of disease, and a readiness and sincerity in the communication of his experience in similar cases, which never failed to secure the confidence of the practitioner at whose recommendation he had been called to the attendance. He had moreover the enviable quality of observing the most honourable conduct towards the gentlemen in attendance with him, without a compromise of the duty he owed to the invalid. He directed the treatment without arrogating to himself any merit for its success, and assisted the efforts of his junior without lessening towards him the confidence of the patient. His punctuality to his appointments rendered professional intercourse with him peculiarly satisfactory; and even when much debilitated by disease, and engaged in a most extensive practice, he would be as punctual in his appointments with the youngest member of the profession as with the eldest, treating each with the same polite consideration. Such is the language applied to the celebrated Dr. Cleyne.

No man enjoyed the tranquil pleasures of social life with a greater zest than Dr. Huntt; and none ever contributed more to them by amenity of temper, kindness of heart, general intelligence, and sound judgment. His company was always welcome in a circle of either sex, and notwithstanding a certain abruptness of manner, something like that attributed to the celebrated Abernethy, he soon became a favourite with the young and old, the grave and the gay. The loss of such a man, it may easily be imagined, will be long and deeply deplored in the society where he *dwelt.*

